# Information technology acceptance and adoption in the telemedicine sector: a case of Mobile health apps

**DOI:** 10.3389/fdgth.2026.1718447

**Published:** 2026-02-16

**Authors:** Bareeq AlGhannam, Mariam Alsabah, Shaikhah Alainati, Ahmad Alsaber, Faisal AlReshaid, Omar AlHussainan, Omer Gibreel, Mohammad Alkandari

**Affiliations:** 1 Computer Department, College of Business Studies, The Public Authority for Applied Education and Training, Kuwait City, Kuwait; 2 Finance Department, College of Business and Entrepreneurship, Abdullah Al Salem University, Kuwait City, Kuwait; 3 Management Department, College of Business Studies, Public Authority for Applied Education and Training (PAAET), Kuwait City, Kuwait; 4 Management Department, College of Business and Economics, American University of Kuwait, Kuwait City, Kuwait; 5 Center for Financial Studies, UniSA Business, University of South Australia, Adelaide, SA, Australia; 6 College of Business, Department of Management Information Systems, Gulf University for Science and Technology, Kuwait City, Kuwait; 7 Faculty of Health & Social Sciences, Bournemouth University, Bournemouth, Dorset, United Kingdom

**Keywords:** healthcare service, Kuwait, mobile health application, pharmacist, technology acceptance

## Abstract

**Background:**

Pharmaceutical care can be improved via mobile health (mHealth) applications (apps); however, the engagement of these apps depends on healthcare providers' acceptance. Thus, identifying barriers and facilitators to utilizing mHealth apps is needed to develop and facilitate their use.

**Objective:**

The objective of this research was to investigate the various elements that influence the acceptability and utilization of mHealth apps in the delivery of pharmaceutical care services, as perceived by pharmacists working in the government health sector of Kuwait.

**Methods:**

A cross-sectional survey was conducted, and the results were used in the mobile health technology acceptance model (m-TAM) to measure the behavioral intentions of pharmacists with regard to the acceptance and use of mHealth apps.

**Results:**

Multiple elements influence the behavioral intention of pharmacists to use mHealth apps, including Compatibility (CO), Performance Expectancy (PE), Personal Innovativeness (PI) and Effort Expectancy (EE) were statistically significant predictors. The mediating role of PE was found to be statistically significant in the link between CO and BI and in the association between EE and BI. The substantial mediation effects of Effort Expectancy (EE) and Performance Expectancy (PE) were seen in the three associations between job-related mental demands (MA) and burnout (BI), professional identity (PI) and burnout (BI), and perceived social support at work (PSA) and Behavioural Intention (BI). The results further demonstrate that EE substantially mediates the connections between MA and PE, MSE and PE, PI and PE, and PSA and PE.

**Conclusions:**

These results suggest that the acceptance of mHealth apps by pharmacists in Kuwait is multifaceted and requires evaluating a chain of effects. As a result, developing apps that provide pharmaceutical care requires consideration of each one of these factors. Results can direct policymakers and stakeholders in their efforts to implement mHealth in Kuwait.

## 1 Introduction

### 1.1 Background

The rapid integration of mobile health (mHealth) technologies is revolutionizing healthcare delivery, offering unprecedented opportunities to enhance patient care, improve clinical workflows, and expand access to medical services (1). As a key component of telemedicine, mHealth utilizes mobile devices such as smartphones and tablets to support a wide range of health-related functions, from remote patient monitoring and medication management to health education and clinical communication (2). The global adoption of these technologies has been accelerated by their potential to address critical challenges in healthcare. Systems including rising costs, aging populations, and increasing prevalence of chronic diseases (3). In this context, understanding the factors that influence healthcare professionals' acceptance and use of M health applications is crucial to successful implementation and for realizing the full benefits of digital health transformation (4, 5).

Pharmacists, as integral members of the healthcare team, are increasingly expected to leverage digital tools to optimize for medical care; their roles are expanding beyond traditional dispensing to include medication therapy management, patient counseling, and chronic disease management, all of which can be significantly enhanced by mHealth technologies (6, 7). However, the adoption of these tools is not automatic and is influenced by a complex interplay of behavioral, organizational, and technological factors (8). The Technology Acceptance Model (TAM) and its variants, such as the Unified Theory of Acceptance and Use of Technology UTAUT and the Mobile Health Technology Acceptance Model (m-TAM) provide robust frameworks for investigating these factors. factors (4, 9). These models posit that perceived usefulness and perceived ease of use are primary determinants of an individual's intention to use a new technology, shaped by external variables and individual characteristics.

In the context of the Middle East and specifically the Gulf Cooperation Council (GCC) countries, governments are making substantial investments in digital health infrastructure to modernize their healthcare systems (10, 11). Kuwait, through its ambitious Kuwait Vision 2035, has identified digital transformation as a cornerstone of its national development strategy, with a particular focus on strengthening the quality and efficiency of its health care sector (12, 13). This vision includes the nationwide implementation of Electronic Health Records (EHR) systems and the promotion of digital health services to both public and private providers (14). Although the Minister of Health (MOH) has introduced several digital health initiatives, such as electronic perception and documentation systems. There is currently no unified national M health application designed specifically for pharmacists. Instead, adoption has relied on limited hospital or department-level pilots, resulting in inconsistent exposure and fragmented use across Mohs facilities. The gradual integration of mobile-supported pharmacy workflows began around 2017. Yet, implementation remains uneven, leaving many pharmacists without standardized access to mobile-enabled tools. This fragmented landscape also reflects broader challenges, including variable infrastructure, limited training opportunities, and hesitancy toward shifting from. Deeper or desktop-based processes to mobile-enabled systems.

Mobile health technologies have expanded rapidly across global healthcare systems, yet Kuwait contends to experience fragmented and uneven implementation, particularly within the Ministry of Health pharmacy settings. Pharmacists increasingly perform clinically oriented droughts, but they do so without. Standardized or widely adopted mHealth system to support medication management documentation or patient counselling. This gap between national digital health ambitions and foreign client readiness creates an urgent need to assist pharmacists' acceptance of mobile tools before large-scale deployment occurs. Without understanding the behavioral, organizational, and contextual factors that influence their adoption, future policy decisions risk investing in systems that do not align with pharmacists' needs, workflow realities, or capability levels. This makes an evidence-driven assessment of mHealth acceptance not only timely but essential for Craig's broader digital transformation agenda.

Despite these strategic imperatives, Significant research gaps remain in the literature concerning mHealth adoption in this region. While several studies have explored technology acceptance in healthcare, most have focused on physicians and nurses in North American, European, or Asian contexts (4). There is a notable scarcity of research examining the specific factors that influence mHealth adoption among pharmacists, particularly within the unique government-led health care system of a Gulf country like Kuwait (10). Previous research in the Middle East has highlighted several barriers to telemedicine adoption, including cultural resistance, lack of training, and poor infrastructure, but has not adequately addressed the perspectives of pharmacists or the challenges of implementing mHealth within a centralized public health care system (15, 16). Furthermore, few studies have applied a comprehensive theoretical model like the m-TAM to investigate the interplay of individual, technological, and social factors influencing adoption in this specific demographic and organizational context.

This study aims to address these gaps by offering several novel contributions to the literature. First, it provides one of the first comprehensive empirical analyses of mHealth acceptance among government-employed pharmacists in Kuwait, a critical but under-researched group of healthcare professionals. Second, by applying and extending the m-TAM, this research offers context-specific insights into the behavioral, motivational, and contextual factors that shape technology adoption within a centralized public healthcare system undergoing rapid digital transformation. Third, the study's findings on the mediation and moderation effects of variables. Such as personal innovativeness. Mobile self-efficacy and gender provide a more granular understanding of the adoption process than previously available for this population. Ultimately, this research generates practical, evidence-based recommendations for policymakers, hospital administrators, and technology developers seeking to foster the effective integration of mHealth technologies into pharmacy practice in Kuwait and similar settings.

### 1.2 Theoritical framework

This study adopts the Mobile Health Technology Acceptance model (TAM) to investigate the factors influencing pharmacists’ behavioral intention to use m-health applications in Kuwait. The M term is an adaptation of the original technology acceptance model term tailored to the specific context of mobile technologies. It provides A robust framework for understanding user acceptance by integrating core TAM constructs with additional variables relevant to the mobile environment. This section discusses the theoretical foundation of each construct, including the proposed model. Supported by evidence from recent literature in telemedicine and m-health adoption, the following table provides the operational definitions of each variable (see Table 1), followed by a discussion of their theoretical relevance and application in recent research.

**Table 1:** Overview of key constructs in technology acceptance models: definitions and theoretical foundations.

Variable	Operational definition
Behavioral intention (BI)	The user's subjective likelihood of using mHealth applications in their professional practice.
Effort expectancy (EE)	The degree of ease associated with the use of the mHealth system.
Performance expectancy (PE)	The degree to which an individual believes that using the mHealth system will help them attain gains in job performance.
Social Influence (SI)	The degree to which an individual perceives that important others believe they should use the new system.
Compatibility (CO)	The degree to which an innovation is perceived as being consistent with the existing values, needs, and past experiences of potential adopters.
Personal INNOVATIVENESS (PI)	The willingness of an individual to try out any new information technology.
Mobile anxiety (MA)	An individual's apprehension or fear when faced with the possibility of using mobile technology.
Mobile self-efficacy (MSE)	An individual's belief in their ability to successfully use mobile technology to accomplish specific tasks.
Result demonstrability (RD)	The tangibility of the results of using an innovation.
Perceived service availability (PSA)	The user's perception of the availability and quality of supporting infrastructure for the mHealth service.
Technical support and training (TST)	The availability of organizational and technical infrastructure to support the use of the system.

Definitions synthesized from major technology acceptance frameworks, including TAM, UTAUT, DOI, and m-TAM.

### 1.3 Study constructs

Behavioral Intention (BI) is the central dependent variable in most technology acceptance models, representing the most direct predictor of actual system use (17). In the context of healthcare, a professional's intention to use technology is a critical precursor to its successful integration into clinical practice. Numerous meta-analyses and systematic reviews have consistently confirmed BI as the primary outcome variable for assisting the Acceptance of various health technologies, including EHRs, Telemedicine platforms, and mHealth apps (4, 18).

Performance Expectancy (PE), equivalent to perceived usefulness in the original TAM, is one of the most powerful predictors of behavioral intention (4). It reflects the user's belief that the technology will enhance their job performance. In healthcare, this could mean improved efficiency, better access to information. Or enhanced patient care quality. Recent studies on telehealth and m-health adoption among healthcare professionals have consistently found a strong positive relationship between PE and the intention of using technology, confirming its critical role in motivating adoption (2, 18).

Effort Expectancy (EE), or Perceived Ease of Use, refers to how effortless a user perceives a technology to be. If pharmacists believe an mHealth app is intuitive and easy to navigate. They are more likely to perceive it as useful and consequently develop a stronger intention to adopt it (4). The relationship between EE and PE, and EE and BI, is one of the most well-established findings In technology acceptance literature, holding across diverse healthcare settings and technologies (18, 19).

Social Influence (SI) captures the impact of the social environment, including the opinions of colleagues, supervisors, and the wider organization. In a hierarchical setting, like a government ministry, the perceived expectations of peers and superiors can significantly shape an individual's adoption decisions (18). Recent research confirms that SI is a significant predictor of intention to use health IT, particularly in the early stages of implementation, when users may rely on social cues to form their attitudes (9).

Compatibility (CO) is a key construct from the Innovation Diffusion Theory IDT that has been integrated into many acceptance models. It measures the alignment of technology with a user's existing work practices, values, and needs. For pharmacists, an mHealth app is more likely to be adopted. If it integrates smoothly with their clinical workflow and is perceived as consistent with their professional role (20). Studies in telemedicine have shown that high compatibility reduces resistance and facilitates adoption by ensuring the technology fits the user's context.

Personal Innovativeness (PI) reflects an individual's inherited trait of being open to trying new technologies. Individuals with higher PI are more likely to have positive beliefs about new systems and are less resistant to change (38). In the context of mHealth, a pharmacist's personal innovativeness can directly influence their intention to adopt new digital tools and may also moderate the effects of other TAM variables. This construct is particularly relevant in the fast-evolving digital health landscape (21).

Mobile Anxiety (MA) and Mobile Self-Efficacy (MSE) are two critical psychological constructs that address a user's confidence and comfort with mobile technology. MA refers to the fear or apprehension associated with using the technology, while MSE is the user's belief in their own ability to use it effectively. Research has shown that higher anxiety acts as a barrier, while higher self-efficacy is a significant facilitator of M health adoption (9, 22). MSE, in particular, has been found to have both direct and indirect effects on behavioral intention by positively influencing performance and effort expectancies (9).

Result Demonstrability (RD), Another construct from IDT refers to the visibility and communicability of a technology's benefits. The positive outcomes of using an mHealth app, such as time savings or improved patient outcomes, are tangible and easily observable; adoption is more likely (23). This is because demonstrable results provide concrete evidence of the technology's value, reinforcing performance expectancy and encouraging wider adoption within the organization (20).

Perceived Service Availability (PSA) and Technical Support and Training (TST) are organizational facilitators that are crucial for technology adoption in any institutional setting. PSA relates to the reliability of the underlying infrastructure (e.g., network connectivity), while TST concerns the availability of help and training Resources. In the context of Kuwait's MOH, where digital systems are being implemented at a large scale, the presence of robust Technical Support and comprehensive training is essential to mitigate user anxiety, enhance self-efficacy, and ensure that pharmacists can use mHealth tools effectively and efficiently (14, 24). The lack of such support has been identified as a major barrier to telemedicine adoption in the Middle East (2).

## 2 Methodology

### 2.1 Study overview

**Figure 1:**
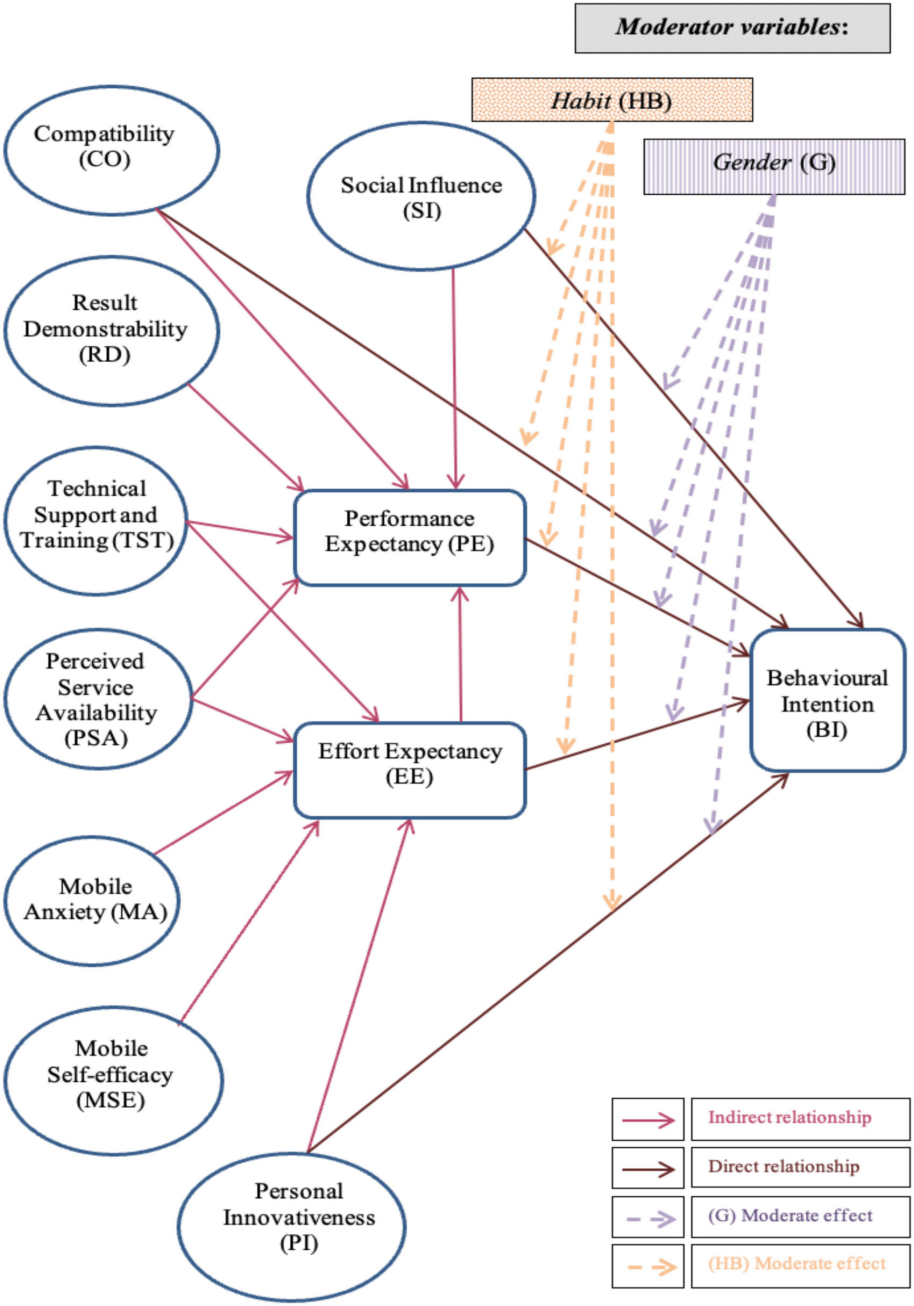
Model for investigating factors of pharmacists’ acceptance of using mHealth apps.

This study is a quantitative research study that implemented a cross-sectional survey to obtain the necessary inputs and apply the mobile health technology acceptance model (m-TAM) to assess the behavioral intention of pharmacists in Kuwait regarding the use of mHealth applications as it shown in Figure 1. The research was conducted after obtaining ethical permission from the Ethics Committee of Bournemouth University's Faculty of Health and Social Science and the Head of the Standing Committee for the Coordination of Medical Research at the MOH. All participants supplied informed consent.

### 2.2 Study design

The required sample size was calculated based on a target population of 2,476 Pharmacists at a 95% confidence level, and a 5% margin of error. This yielded a minimum required sample size of 333 participants. The final data set included 440 complete responses exceeding the required threshold. Before analysis, the data set was cleaned to remove complete responses. Surveys with more than 10% missing data were excluded, and all the subsequent analyses were performed using listwise deletion to ensure consistency across constructs.

### 2.2 Sampling method

The target population was Kuwaiti MOH-affiliated pharmacists. Pharmacists registered with the Kuwait Pharmacy Society (KPS) and working in Kuwait's government healthcare system, irrespective of gender, position, experience, or nationality, were contacted by email or phone. According to KPS records, 5,415 pharmacists were registered. Of these, 2,476 were government pharmacists working in hospitals, primary care facilities, and administrative work centres.

#### 2.2.1 Inclusion and exclusion criteria

Pharmacists were included if they were currently employed by the MOH and had at least six months of clinical or administrative experience using any digital or electronic documentation system. Pharmacists working in the private sector, pharmacy technicians, student interns, or individuals with no exposure to electronic documentation workflows were excluded from the study. This ensured that participants had sufficient familiarity with mobile- or electronic-enabled processes to meaningfully evaluate mHealth acceptance.

### 2.3 Data collection

To obtain inputs for the m-TAM, each participant completed a survey in Arabic or English (according to participants’ preferences) for which the survey items were adopted from the literature. A skilled translator translated the survey into Arabic from English. Because the research team's supervisory staff speaks Arabic and English, they evaluated the back-translations to verify correct wording throughout the survey and conducted multiple reviews.

Although Arabic is the national language, English is the operational language of pharmacy practice in Kuwait, and medication systems, electronic documentation, and clinical decision-support tools are all maintained in English. The questionnaire therefore, remained in English to preserve the validity of previously validated instruments and to accommodate expatriate pharmacists who commonly use English in professional settings.

In addition to translation and back-translation, several steps were taken to ensure the validity and reliability of the survey instrument across cultural and linguistic contexts. First, content validity was addressed by adapting items from validated instruments previously applied in mHealth and technology acceptance studies, notably (25). Following translation, cognitive interviews were conducted with six pharmacists affiliated with the MOH to assess item clarity, cultural appropriateness, and alignment with domain-specific language. These participants reflected a range of job roles and experience levels within the public sector. Based on this feedback, minor modifications were made to improve clarity and reduce ambiguity. Subsequently, A pilot test was conducted with a sample of 25 pharmacists. Pharmacists who participated in the pilot were not included in the final survey to avoid potential bias resulting from prior exposure to the questionnaire. Results from the pilot were used to assess item performance, language flow, and technical usability of the online and paper formats. This process led to adjustments in formatting, layout, and question sequencing. The final instrument, consisting of 34 items across 11 constructs, was psychometrically refined using both classical test theory criteria (e.g., item-total correlations) and structural equation modeling criteria (i.e., convergent validity, discriminant validity, composite reliability). During the full study analysis phase, the demographic section of the survey included an item for the participant's job title, which was phrased as “What is your current professional position within the MOH?” The response options were based on standardized job classifications used within Kuwait's MOH system (e.g., “Pharmacist,” “Senior Pharmacist,” “Pharmacy Specialist,” etc.). These titles reflect formal civil service designations. Although expatriate Pharmacists are part of the MOH workforce, Nationality was intentionally not collected to avoid identifiability concerns and because nationality doesn't influence pharmacists' access to or interaction with digital systems, which follow standardized workflows across all MOH facilities.

### 2.4 Survey distribution approach

To ensure broad participation across the diverse body of pharmacists employed by the MOH, a multimodal recruitment strategy was adopted:

Direct Contact via Kuwait Pharmacy Society (KPS): Pharmacists were contacted through the official. E-mail and telephone channels.

Social Media Dissemination: The survey link was shared through pharmacist-focused professional groups.

Physical Distribution in MOH Facilities: Paper surveys were distributed through pharmacy department heads who verified staff eligibility.

This combined strategy was necessary because no signal channel reaches all government pharmacists, and work patterns vary significantly.

To maintain data integrity:

A screening item confirmed MOH employment,
Online submissions were restricted to one per IP address,
Participants were instructed not to complete the survey more than once,
Physical responses were manually entered and cross-checked to remove duplicates.

#### 2.4.1 Online and offline mode justification

Both online and paper-based surveys were used due to the diverse working conditions within MOH facilities. Many pharmacists work rotating or extended shifts, limiting computer access. Others are stationed in facilities with restricted digital access, offering online. Offline options increased inclusivity, accessibility and response rates.

#### 2.4.2 Online survey platform

The online survey was administrated using Google Forms, which allowed secure, user-friendly access from both mobile devices and computers.

### 2.5 Survey instrument validation

To ensure the validity and reliability of the instrument across languages and cultural contexts, a multi-step validation process was undertaken.

Content validity: Ensured by adopting constructs and items from previously validated instruments in the literature (notably^18^).

Translation process: Include this forward and backward translation conducted by bilingual experts, followed by iterative reconciliation.

Cognitive testing: Conducted through a cognitive interview with six MOH-affiliated pharmacists to assist. Got some clarity, cultural appropriateness, and comprehension.

Pilot testing: A pilot study with the 25 pharmacists was conducted before full data collection. Feedback was used to refine item wording, eliminate ambiguity, and adjust formatting for both Arabic and English versions.

Psychometric assessment: Beyond the PLS-SEM threshold for reliability and validity AVE, Cronbach's alpha, composite reliability, Fornell-Larcker), We also examined item total correlations and factor loadings during the pilot to ensure construct coherence.

### 2.6 Mobile health technology acceptance model (m-TAM)

All constructs in the m-TAM were measured using established items adapted from prior technology acceptance studies. A five-point Likert scale ranging from 1 (strongly disagree) to 5 (strongly agree) was used for all items. Performance expectancy, effort expectancy, and social influence were measured using items reflecting perceived usefulness, ease of use, and social pressures. Compatibility, personal innovativeness, mobile self-efficacy, mobile anxiety, result demonstrability, and perceived service availability were measured using items capturing contextual and individual determinants. Behavioral intention was assessed through items evaluating pharmacists' willingness and likelihood to adopt mHealth applications in their professional practice.

### 2.7 Data analysis

Demographics for the participants were assessed and presented as descriptive statistics (frequencies and percentages). m-TAM model used the survey results (Likert scales outcomes) to measure the impact of each independent and mediating variable towards the dependent variables using partial least squares structural equation modelling (PLS SEM). PLS SEM is useful when there are complex causation paths to be evaluated (26). PLS SEM enabled the study to do measure path coefficients and assessment of the model's quality in hypothesis testing without considering moderating pathways (27).

The PLS SEM has two sub models, namely i) measurement model and ii) structural model (26). Measurement model highlights the relationship between the empirics and the latent variable to ensure the to ensure the model is capturing the data. The structural model on the other hand, shows the holistic web of relationships represented in the model among the laten variables. Both the measurement as well as structural models are evaluated. In general, if the measurement model fails to meet the minimum requirements for reliability and validity, the assessment of the structural model has no value (28). As such, the model was validated and interpreted to ensure reliability. The assessment of convergent validity was conducted in order to ascertain the degree of association between the measurements within each variable using the Average Variance Extracted (AVE), Cronbach's alpha, and composite reliability. An AVE threshold of .50 was set, which is the preferred criterion for establishing convergent validity as suggested by the seminal work on PLS SEM (29).

Discriminant validity of the model, which refers to the extent to which constructs exhibit distinctiveness, indicating their differentiation from other items under analysis, was also assessed (30). The present study employed three criteria to assess discriminant validity: the Fornell–Larcker criterion, cross-loadings, and the heterotrait–monotrait ratio (26, 31). Based on the criterion established by Fornell and Larcker (29), it is expected that the square roots of the AVE values should exceed the correlations between the components. A threshold of .85 was used for the heterotrait–monotrait ratios (26).

Goodness of fit was assessed using the coefficient of determination, denoted as *R*^2^, a statistical metric used to assess the quality of a model's fit. It quantifies the proportion of variability in latent variables that can be attributed to the corresponding indicators. Corresponding indicators are the questions used in the questionnaire. In essence, the coefficient of determination demonstrates the degree to which the independent variables of a given model elucidate the dependent (outcome) variable. The coefficient of determination, *R*^2^, exhibits a range of values from 0 to 1. *R*^2^ value of .19 signifies a weak influence, .33 signifies a moderate influence, and .67 signifies a significant influence (31).

Structural model assessment was conducted to assess the hypothesis (direction of variable influence) inherent within the m-TAM. The structural model analysis include the assessments of both independent as well as mediating variables. The path coefficient analysis and model quality evaluation in this research indicated causal linkages between variables. Path analysis indicates that indicators can directly or indirectly affect a hidden variable or outcome variable. Path analysis uses mediation to quantify indirect correlations between variables (31). Mediation analysis elucidates the effect of an independent variable on an outcome by considering an intermediary variable, referred to as a mediator (32).

The moderating effect of habit and gender was investigated by determining its interaction effect on the relationship between the effects of PE, EE, SI, PI, and compatibility on the dependent variable, BI. The moderating effect of gender was also investigated by determining its interaction effect on the relationships between the effects of PE, PI, compatibility, EE, and SI on the main dependent variable, BI.

The data were analysed using both IBM's Statistical Package for the Social Sciences version 28 and SmartPLS V.4.0.9.6, the latter specifically for performing partial least squares structural equation modelling (31). SmartPLS was used for structural equation modelling for four reasons: (1) it is the best method for developing existing theory, (2) it is recommended for studies involving complex models, (3) it allows analyzing each model as a single block instead of splitting it into separate pieces, and (4) it allows more accurate estimations, providing parallel analysis for both the measurement and structural models at the same time (31).

### 2.8 Ethical considerations

Participants didn't receive any financial incentives, gifts, or compensation for completing the survey. Participation was entirely voluntary, unanimous, and conducted in accordance with approved ethical procedures.

## 3 Results

### 3.1 Demographic characteristics analysis and sample size

The demographic profile of the 440 participant pharmacists from Kuwait's Ministry of Health provides essential context for interpreting the study's findings on M health adoption presented in Table 2. The sample is characterized by a majority of female respondents (62.5%), A significant concentration of participants in the 26–35 Age group (45.2%), And a high level of educational attainment, with most holding a bachelor's degree in pharmacy (85.9%). These characteristics are not merely descriptive but offer analytical insights when viewed through the lens of technology acceptance literature. The predominance of pharmacists in the 26–35 age Range is particularly noteworthy. This demographic, often comprising digital natives, is generally associated with higher levels of technological literacy and greater personal innovativeness (33). Younger healthcare professionals have been shown to exhibit a more positive attitude toward adopting new technologies, including M Health, as they are more accustomed to integrating digital tools into their daily lives and professional workflows (34). This demographic profile may contribute to the relatively high levels of behavioral intention observed in the study, as this age group is typically more receptive. To the benefits of mobile technology in enhancing Job performance and efficiency. Furthermore, the samples gender distribution with a female majority aligns with the pharmacy workforce demographics in many parts of the world, including the Middle East. While some technology acceptance studies have reported gender as a significant moderator of adoption behavior, often with males showing higher acceptance. Recent meta-analysis in the health care contexts. I found these effects to be inconsistent or non-significant (18, 35). For instance, a major 2025 meta-analysis by Yang et al. found no significant moderating effect of gender on the core relationships within technology acceptance models in healthcare (18). This suggests that professional Rd. organizational context and the perceived value of the technology may be more influential determinants of adoption than gender alone. The findings in our study that gender emerged as a meaningful moderator highlight a context-specific nuance, Warranting A deeper discussion of how gender roles and expectations within the Kuwaiti healthcare system might interact with technology adoption. The high level of education among respondents is another critical factor. A highly educated workforce is more likely to possess the cognitive skills and. Efficacy is required to learn and adapt to new technologies (9). Pharmacists with advanced training are also more likely to appreciate the value of evidence-based practice and recognize the potential of mHealth applications to provide rapid access to clinical information. Thereby improving patient care. This educational background likely enhances this performance expectancy and reduces mobile anxiety, creating a more favorable disposition toward mHealth adoption. In summary, the demographic characteristics of the sample provide a foundation and understanding of the user group. The concentration of young, highly educated pharmacists suggests a population that is likely predisposed to technology adoption. However, the interplay of these characteristics with the specific cultural and organizational context of Kuwait's public healthcare system creates a unique environment for mHealth implementation. This analytical interpretation, supported by contemporary research, moves beyond simple description to offer a richer understanding of the factors shaping user acceptance patterns in this specific telemedicine context.

**Table 2:** Demographic details of the quantitative study's participants (*N* = 440).

Question		*n*	%
Would you please specify your gender?	Man	165	37.5%
	Woman	264	60.0%
	Prefer not to say	11	2.5%
Would you please select your age category?	<30 years	97	22.0%
	30–39 years	201	45.7%
	40–49 years	102	23.2%
	50–59 years	24	5.5%
	≥60 years	7	1.6%
	Prefer not to say	9	2.0%
Would you specify your highest level of education attained?	Bachelor's degree in pharmacy	385	87.5%
	PharmD or clinical pharmacy degree	20	4.5%
	Master's degree	29	6.6%
	M.Phil. or PhD degree	6	1.4%
What type of institution are you currently working in?	MOH general hospital	212	48.2%
	MOH specialist hospital	27	6.1%
	MOH primary care centre	134	30.5%
	Central Medical Stores Administration	23	5.2%
	Drug Inspection Administration	2	0.5%
	Pharmaceutical & Herbal Medicines Registration & Control Administration	8	1.8%
	Other	34	7.7%
What is your current professional position within the MOH?	Junior pharmacist	68	15.5%
	Pharmacist	143	32.5%
	Pharmacy specialist	70	15.9%
	Senior pharmacist	80	18.2%
	Senior pharmacy specialist	52	11.8%
	Head of pharmacy specialist	27	6.1%
How long have you been working as a pharmacist, including years as a beginner pharmacist?	<5 years	105	23.9%
	5–10 years	118	26.8%
	11–15 years	110	25.0%
	16–19 years	54	12.3%
	≥20 years	53	12.0%

Titles listed reflect formal Ministry of Health employment classifications in Kuwait.

### 3.2 Convergent validity

The AVE values fell within the range of .62–.82, surpassing the threshold of .50 (see Table 3).

**Table 3:** Reliability and validity of the constructs.

Variable	Cronbach's *α*	rhoA	Composite reliability	Average variance extracted (AVE)
BI	.886	.892	.929	.814
CO	.889	.894	.931	.819
EE	.868	.869	.919	.791
MA	.858	.890	.912	.776
MSE	.840	.849	.904	.758
PE	.882	.884	.918	.738
PI	.812	.825	.888	.726
PSA	.864	.869	.917	.786
RD	.709	.731	.872	.773
SI	.808	.869	.868	.622
TST	.879	.892	.925	.804
Criteria	>.7	>.7	>.7	>.5

BI, behavioural intention; CO, compatibility; EE, effort expectancy; MA, mobile anxiety; MSE, mobile self-efficacy; PE, performance expectancy; PI, personnel innovativeness; PSA, perceived service availability; RD, result demonstrability; SI, social influence; TST, technical support and training.

### 3.4 Discriminant validity

Table 4 presents the results of the Fornell–Larcker criterion. The square root of all AVE values, represented by the diagonal elements, were higher than their respective correlations with other constructs.

**Table 4:** Results for the Fornell–Larcker criterion.

Construct	BI	CO	EE	MA	MSE	PE	PI	PSA	RD	SI	TST
BI	**.902**										
CO	.559	**.905**									
EE	.513	.497	**.889**								
MA	−.282	−.240	−.297	**.881**							
MSE	.594	.484	.454	−.163	**.871**						
PE	.525	.535	.628	−.217	.461	**.859**					
PI	.572	.527	.473	−.187	.466	.429	**.852**				
PSA	.558	.515	.512	−.259	.537	.441	.470	**.887**			
RD	.512	.524	.499	−.298	.437	.401	.637	.475	**.879**		
SI	.329	.344	.271	−.020	.311	.317	.327	.295	.228	**.789**	
TST	.525	.398	.453	−.212	.538	.390	.416	.650	.419	.283	**.896**

The bold diagonal element represents the square root of each construct's average variance extracted. BI, behavioural intention; CO, compatibility; EE, effort expectancy; MA, mobile anxiety; MSE, mobile self-efficacy; PE, performance expectancy; PI, personnel innovativeness; PSA, perceived service availability; RD, result demonstrability; SI, social influence; TST, technical support and training.

### 3.5 Structural model assessment

The findings of the path coefficient analysis are presented in Table 5. Five of the 16 hypotheses examined were unsupported, and 11 were supported. The variables of CO, PE, PI, and EE were statistically significant associated (*p* < .05) with Behavioural Intention (BI). Forty-seven per cent (*R*^2^_adj_ = .47) of the variation in BI was explained by CO, PE, PI, and EE. The effects of CO, SI, and EE on PE were statistically significant (*p* < .05), whereas the direct effects of PSA, RD, and TST on PE were not (*p* > .05). Beyond that, CO, SI, and EE could explain 47% of the variance in PE. The direct effect of MA, MSE, PI, and PSA on EE was significant, while the direct effect of TST on EE was not (*p* > .05). The *R*^2^_adj_ value shows that MA, MSE, PI, and PSA could explain 37% of the variation in EE. As shown in Table 4, *f*^2^ scores of .02, .15, and .35 show small, medium, and large effect sizes, respectively. The f^2^ scores justify the effect size varying from small to medium in the model.

**Table 5:** Path coefficients and assessment of the model's quality in hypothesis testing without considering moderating effects (direct effect).

Hypothesis	Original sample (O)	Sample mean (*M*)	Standard deviation (*SD*)	*t* statistics (|O/*SD*|)	*p*	Decision	*f* ^2^	VIF	*R* ^2^_adj_
CO → BI	.221	.221	0.070	3.164	.002	Accept	.054	1.725	.469
PE → BI	.173	.172	0.054	3.194	.001	Accept	.030	1.889	
PI → BI	.294	.290	0.060	4.928	.000	Accept	.106	1.551	
SI → BI	.065	.067	0.036	1.801	.072	Reject	.007	1.196	
EE → BI	.138	.143	0.065	2.136	.033	Accept	.020	1.845	
CO → PE	.253	.256	0.063	4.026	.000	Accept	.070	1.722	.464
PSA→ PE	.030	.028	0.064	0.467	.641	Reject	.001	2.124	
RD → PE	−.007	−.006	0.048	0.155	.877	Reject	.000	1.615	
SI → PE	.089	.091	0.038	2.371	.018	Accept	.013	1.177	
TST → PE	.045	.048	0.057	0.793	.428	Reject	.002	1.830	
EE →PE	.446	.441	0.069	6.458	.000	Accept	.229	1.644	
MA → EE	−.152	−.153	0.050	3.033	.002	Accept	.035	1.080	.373
MSE→ EE	.150	.147	0.059	2.536	.011	Accept	.022	1.644	
PI → EE	.229	.230	0.052	4.415	.000	Accept	.060	1.416	
PSA → EE	.217	.218	0.059	3.684	.000	Accept	.037	2.021	
TST → EE	.104	.106	0.057	1.823	.069	Reject	.009	1.914	

VIF, variance inflation factor; BI, behavioural intention; CO, compatibility; EE, effort expectancy; MA, mobile anxiety; MSE, mobile self-efficacy; PE, performance expectancy; PI, personnel innovativeness; PSA, perceived service availability; RD, result demonstrability; SI, social influence; TST, technical support and training

### 3.6 Mediation analysis

Table 6 presents a visual representation of the indirect impacts of the independent variables on the dependent variables. The mediating role of PE was found to be statistically significant in the link between CO and BI and in the association between EE and BI. The substantial indirect effects of effort expectancy (EE) and performance expectancy (PE) were seen in the three associations between mobile anxiety (MA), behavioural intention (BI), personal innovativeness (PI) and perceived service availability (PSA). The results further demonstrate that EE substantially mediates the connections between MA and PE, MSE and PE, PI and PE, and PSA and PE.

**Table 6:** Path coefficients and assessment of the model's quality in hypothesis testing considering moderating effects.

Hypothesis	Original sample (O)	Sample mean (*M*)	Standard deviation (*SD*)	*t* statistics (|O/*SD*|)	*p*
Gender × CO → BI	0.013	0.011	0.072	0.184	0.854
Gender × EE → BI	0.14	0.138	0.059	2.39	0.017
Gender × PE → BI	−.111	−.110	0.052	2.145	0.032
Gender × PI → BI	−.120	−.111	0.061	1.981	0.048
Gender × SI → BI	0.064	0.06	0.038	1.663	0.097
Habit × CO → BI	0.101	0.089	0.055	1.827	0.068
Habit × EE → BI	0.021	0.027	0.044	0.467	0.641
Habit × PE → BI	−.111	−.100	0.059	1.875	0.061
Habit × PI → BI	0.039	0.037	0.054	0.714	0.475
Habit × SI → BI	−.001	−.003	0.035	0.037	0.971

### 3.7 Analysis of the effects of the moderator variables

**Figure 2:**
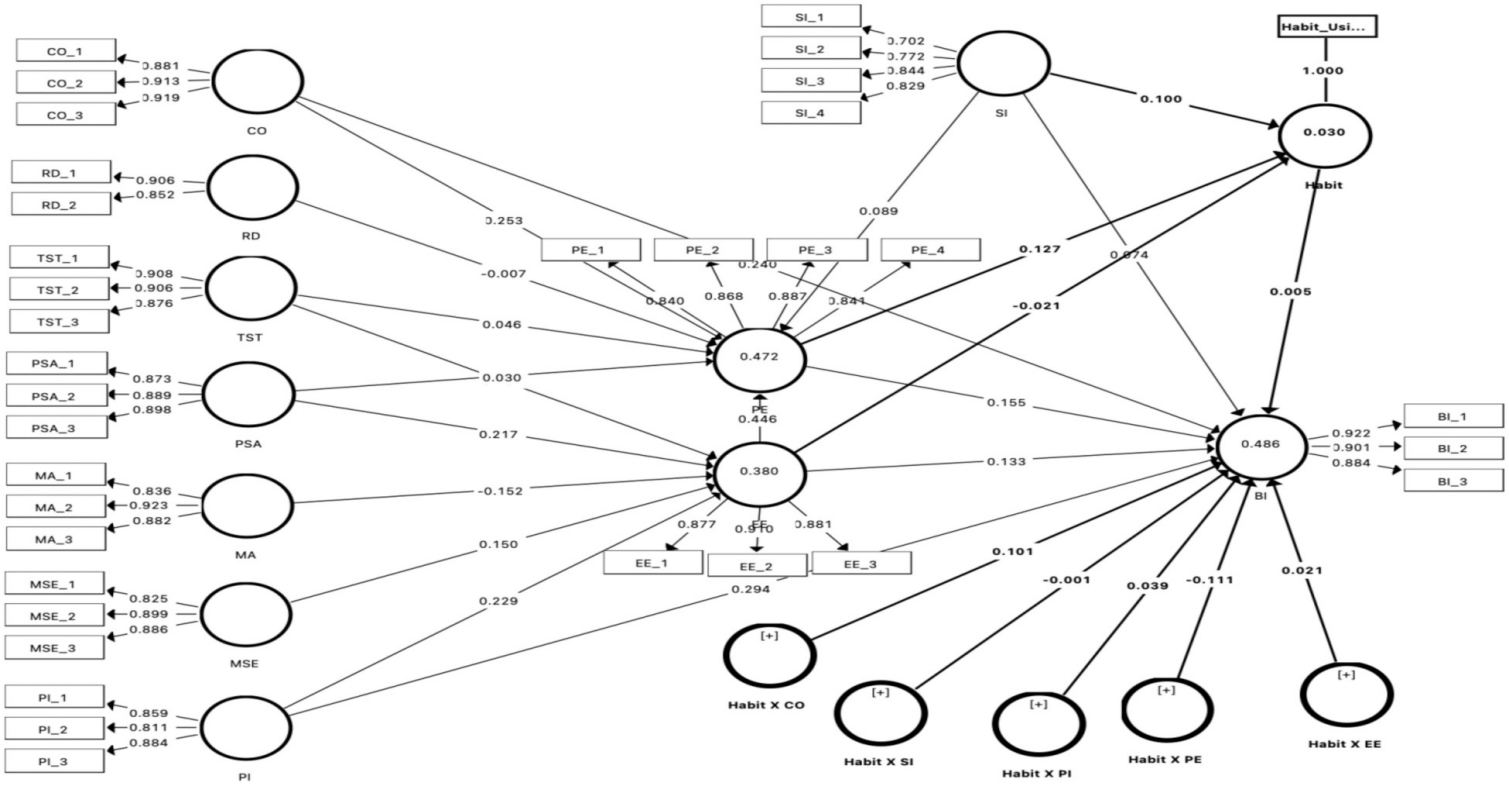
Measurement model (i.e., partial least squares algorithm).

The results demonstrate that habit, as a moderator, did not show any significant effects on the relationships between BI and PE (*β* = –.111, *t* = 1.875, *p* = .061), PI (*β* = .039, *t* = .714, *p* = .475), compatibility (*β* = .101, *t* = 1.827, *p* = .068), EE (*β* = .021, *t* = 0.467, *p* = .641), or SI (*β* = −.001, *t* = .037, *p* = .971), as shown in Figure 2. The moderating effect of habit increased the *R*^2^ value from .475 to .486 in the model. The results demonstrate that gender, as a moderator, had a significant effect on the relationships between BI and PE (*β* = −.111, *t* = 2.145, *p* = .032), PI (*β* = − 0.120, *t* = 1.981, *p* = .048), and EE (*β* = .140, *t* = 2.390, *p* = .017), as shown in Figure 2. The moderating effect of gender increased the *R*^2^ value from .475 to .508 for the model.

## 4 Discussion

This study provides one of the first comprehensive examinations of pharmacists' acceptance of mobile health (mHealth) applications within the Kuwaiti Ministry of Health. Revealing a complex network of behavioral, cognitive, and contextual factors that shape adoption intentions, the findings largely align with the established tenets of technology acceptance models. Yet they also extend the literature by uncovering context-specific dynamics unique to government-led digital health transformation. The results confirmed that performance expectancy (PE), Effort expectancy (EE), Compatibility (CO), and Personal Innovativeness (PI) Are the strongest determinants of pharmacists' intention to adopt mHealth applications. This is consistent with previous work showing that healthcare professionals are more. Willing to embrace new digital tools when they perceive clear performance benefits, ease of use and alignment with their workflow (3, 4, 17). The patterns observed in this study reaffirmed that even highly functional technologies may face adoption challenges if they are perceived as effortful or do not fit naturally within clinical routines.

The study's structural model further clarifies how these relationships interact variables such as Mobile Self-Efficacy (MSE), Mobile Anxiety (MA), and Perceived Service Availability (PSA) influencing key expectancy beliefs, which in turn shape behavioral intention. This indicates that. Pharmacists' readiness to adopt M health tools is not driven by a single determinant but results from a combination of personal confidence, perceived barriers and organizational support. These findings align with the ongoing research showing that digital health adoption improves when professionals feel capable of using the tools and trust that the required technological infrastructure will be reliable (9, 22).

One of the most novel contributions of this study concerns the moderating role of gender, while recent meta-analysis have suggested that gender differences in technology adoption are diminishing globally (18), The present findings reveal more pronounced variations within the Kuwaiti public healthcare context. This indicates that sociocultural norms and the workplace. Dynamics may shape how pharmacists perceive and respond to digital innovations. Rather than contradicting Global Literature. This result highlights the importance of contextual sensitivity, suggesting that gender responsive training programs, communication strategies, and implementation plans may enhance adoption outcomes and insight that is readily addressed in regional studies (2).

Organizational factors also played a central role. Technical Support and Training (TST) and Perceived Service Availability (PSA) were found to strengthen pharmacists' perceptions of usefulness and ease of use, reinforcing long-standing findings that institutional readiness is critical for digital health success (15). In the context of Kuwait's Vision 2035 Digital Transformation Agenda, these results emphasize that investment in reliable infrastructure, accessible training, and ongoing support is not just a logistical requirement but a fundamental determinant of whether mHealth tools will be adopted in practice. Without such support, even highly beneficial technologies may remain underutilized.

Finally, psychological factors demonstrated a clear influence: Mobile Self-Efficacy Act as a facilitator, while Mobile Anxiety served as a barrier. This alliance with the international evidence shows that health care workers' confidence in mobile technology increases adoption, whereas anxiety limits it (9, 22). These insights highlight the importance of building digital confidence through hands-on training, peer support and gradual exposure, especially in environments transitioning from paper-based to digital systems.

In conclusion, this study deepens the understanding of mHealth. Adoption in the Gulf region by applying the m-TAM to a previously understudied Population. While the results confirm core principles of technology acceptance theory, the findings related to gender moderation, psychological readiness, and organizational support extend current knowledge and offer practical Guidance. For policymakers and healthcare leaders, the message is clear. Successful M health implementations require a comprehensive strategy that integrates user friendly design, compatibility with clinical practice, gender responsive and confidence building training, and robust institutional support. Such an. The approach will ensure that pharmacists are well-positioned to adopt and implement health applications effectively, ultimately strengthening Kuwait's national digital health transformation efforts.

### 4.1 Limitations

This study had two main limitations. Firstly, the results of this study may not apply to pharmacists working in non-MOH settings (e.g., private industry) or be generalizable to pharmacists in other countries. It is because the government sector provides a different context and also different support system and expectancies than the private sector. Similarly, in the regulation of public sectors in other countries may also very and will contribute to the nature of determinants for the mHealth app acceptance. The study may, therefore, need to be carefully compared with other studies before making any generalization.

Secondly, the study also used a cross-sectional survey of Kuwaiti pharmacists. This participants selection may affect the findings' generalizability, especially regarding timing, cultural influence, and sample characteristics (36, 37). It is because the cross-sectional data provides a temporally limited perspective of the mHealth acceptability largely depending upon what health systems are in place at time and therefore cannot be generalize to other times when the systems change and when the professionals have more learning of the system and circumstances to use the system. Future research should consider using longitudinal or experimental designs to better establish temporal dynamics and test the directionality of the hypothesized pathways.

Additionally, the modest response rate (17.8%) may introduce concerns regarding external validity. While this rate aligns with typical response rates for voluntary online surveys targeting healthcare professionals, caution should be exercised in generalizing the findings to all MOH pharmacists in Kuwait. Future studies may benefit from employing incentivized recruitment strategies or mixed-mode survey delivery to improve participation.

Further, the model didn't include demographic covariates such as age, gender, or years of experience as control variables. While moderation effects were tested, the absence of covariate adjustment limits the ability to isolate independent effects of the main predictors on behavioral intention. This constrains the causal interoperability of the findings. Should be addressed in future work.

## 5 Conclusion

This study examined the direct and indirect factors influencing pharmacists' behavioral intention to adopt mobile health mHealth applications within Kuwait's Ministry of Health. Revealing A multidimensional set of behavioral, cognitive, and contextual determinants. The result demonstrated that successful and healthy adoption is shaped not only by pharmacists' perceptions of the technologies, such as compatibility, personal innovativeness, mobile anxiety, mobile self-efficacy, and results demonstrably, but also by the organizational environment, including the availability of Technical Support and training. Call mediators such as effort expectancy, performance expectancy, and social influence further shape behavioral intention, underscoring the importance of designing mHealth tools that are intuitive, beneficial to job performance, and supported by a positive workplace environment. Pharmacists in Kuwait have begun integrating mobile technologies into their daily workflows. Sustained and deliberate efforts will be necessary to strengthen these determinants and support long-term digital health adoption. This study extends the application of the m-TAM to government-employed pharmacists in Kuwait, contributing empirical evidence on the behavioral, motivational, and contextual factors that influence health acceptance in the public healthcare system undergoing digital transformation. The findings also offer. Concrete implications for policymakers and developers. Enhancing perceived usefulness. Improving compatibility with the existing workflow, simplifying user interfaces, and strengthening institutional support can meaningfully accelerate adoption. As pharmacists continue to expand their clinical roles in mediation, management, and patient counselling, aligning mHealth solutions with their professional needs will be essential to improving the efficiency, quality, and. Accessibility of pharmaceutical care across Kuwait's healthcare systems.

## Data Availability

The datasets presented in this study can be found in online repositories. The names of the repository/repositories and accession number(s) can be found below: https://doi.org/10.5281/zenodo.17259866.
